# Black *Lycium barbarum* polysaccharide attenuates LPS-induced intestine damage *via* regulation gut microbiota

**DOI:** 10.3389/fmicb.2022.1080922

**Published:** 2023-01-19

**Authors:** An Yan, Houkang Ding, Junjun Liu, Chongliang Bi, Zhaoqing Han, Zhennan Wang, Shah Nawaz, Yizhao Shen, Shudong Liu

**Affiliations:** ^1^College of Animal Science and Technology, Hebei Agricultural University, Baoding, Hebei, China; ^2^College of Veterinary Medicine/Traditional Chinese Veterinary Medicine, Hebei Agriculture University, Baoding, China; ^3^College of Agriculture and Forestry, Linyi University, Linyi, Shandong, China; ^4^Department of Anatomy, Faculty of Veterinary Science, University of Agriculture, Faisalabad, Pakistan; ^5^Key Laboratory of Healthy Breeding in Dairy Cattle (Co-Construction by Ministry and Province), Ministry of Agriculture and Rural Affairs, Baoding, Hebei, China

**Keywords:** Black *Lycium barbarum*, polysaccharide, LPS, mouse, gut microbiota

## Abstract

*Lycium barbarums* are traditionally used as a homology of medicinal plants in China with a potent role in metabolism and immunomodulation. The current study was performed to explore the attenuation effect and microbiota regulation of *Lycium barbarum* polysaccharide (BLBP) on lipopolysaccharide (LPS)-induced intestine damage in mice. A total of 70 mice were randomly divided into five groups; negative control (GA), LPS (GB), both treated with an equal volume of normal saline, and BLBP treatment groups GC (100 mg/kg), GD (200 mg/kg), and GE (400 mg/kg) *via* gavage for 19 days. On Day 19, mice in groups GB, GC, GD, and GE were treated with 10 mg/kg LPS for 24 h and euthanized to collect intestine samples for pathological examination and microbiota sequencing. The results showed a non-significant difference in body weight gain among the five mouse groups; however, mice in the GC and GE groups showed decreased weight gain. An H&E examination revealed that the integrity of intestinal villi was destroyed by LPS, while BLBP supplement alleviated intestinal damage with an increase in villus height and a decrease in crypt depth. A total of over 59,000, 40,000, 50,000, 45,000, and 55,000 raw sequences were found in groups GA, GB, GC, GD, and GE, respectively. LPS challenge decreased alpha diversity indexes significantly (*p* < 0.05), while a non-significant difference was found between different BLBP treatment groups and the GA group. A total of 8 phyla and 13 genera were found among five mouse groups, and BLBP partly restored the bacterial abundance in mice. LPS changed 282 metabolic pathways in KEGG L2, 77 metabolic pathways in KEGG L3, and 205 metabolic pathways in MetaCyc, respectively. The BLBP-supplemented groups, especially GE, showed reverse effects on those metabolic pathways. The current study revealed that BLBP can effectively decrease intestinal damage through the regulation of intestinal microbiota, which may provide new insights for the prevention of intestinal disease using food and medicine homologous of *Lycium ruthenicum*.

## Introduction

The intestine is an important organ for digestion, absorption, and immunity (Ahern and Maloy, 2020; Li et al., 2023). Any damage to the intestine commonly results in intestinal inflammation. Intestinal microbiota are composed of trillions of microorganisms, including various types of bacteria, eukaryotes, archaea, and viruses (Ahern and Maloy, 2020), which contribute greatly to the host physiology by influencing metabolism and immune modulation and affect mental and neurological functions (Honda and Littman, 2016). In the past, many diseases have been reported with microbiota dysbiosis such as inflammatory bowel diseases (Nishino et al., 2018), allergy (Bunyavanich et al., 2016), and diarrhea (Li et al., 2022).

For several millennia, goji berries or *Lycium barbarums* (red and black goji) have been used traditionally as a homology of medicinal and food plants in China (Stanoeva et al., 2021), which can prevent diseases like diabetes, hyperlipidemia, and hepatitis (Islam et al., 2017). Due to their health benefits and anti-aging properties, goji berries are growing frequently in western countries (Jin et al., 2013). Polysaccharides are commonly known as important functional components of goji, which present biological activities related to metabolism, antioxidant activity, and immunomodulation (Jin et al., 2013). Currently, approximately 90% of commercial goji berries are red *Lycium barbarum* (RLB), which is less expensive as compared to black *Lycium barbarum* (BLB), especially those from the Qinghai Tibetan plateau (Liu et al., 2020). There is an increasing trend in the cultivation of BLB across the world as it has high in polyphenols and is rich in antioxidants and polysaccharide contents than RLB (Ni et al., 2013; Sun et al., 2017). A previous study found that the extracts from BLB had significantly higher antioxidant and anti-inflammatory activities than RLB in lipopolysaccharides-stimulated BV2 microglial cells (Magalhães et al., 2022). Gram-negative bacteria membrane-extracted LPS is commonly known for causing oxidative damage and inflammatory reaction into host (Chen X. et al., 2022). However, information about the effect of black *Lycium barbarum* polysaccharide (BLBP) on LPS-induced intestine damage in mice is limited. Hence, the current study was performed to explore the attenuation effect and microbiota regulation of BLBP on LPS-induced mice.

## Materials and methods

### Black *Lycium barbarum* polysaccharide (blbp) extraction and content determination

Approximately 500 g of BLBP was purchased from Tongren Tang (Nanjing, China), and polysaccharide extraction was performed as described in previous studies (Zhao et al., 2016; Yang et al., 2018). The vacuum-dried BLBP was stored at –20°C for future use. The concentration of polysaccharides was detected by piloting the phenol-sulfuric method as described in a previous study (Liu et al., 2021).

### Animal experiment design

A total of 70, 4-weeks-old Kunming mice with an equal number of male and female animals (average wight of 22 ± 2 g) were purchased from Skyford Laboratory Animal Technology Co., Ltd., China. All the mice were given 3 days to accommodate with the surroundings, and the mice were randomly divided into five groups, namely negative control (GA), lipopolysaccharide (GB), and treatment groups (GC, GD, GE). Mice in groups GC (100 mg/kg), GD (200 mg/kg), and GE (400 mg/kg) were treated by BLBP *via* gavage for 19 days, while mice in groups GA and GB were treated with an equal volume of normal saline. On Day 19, mice in groups GB, GC, GD, and GE were treated with 10 mg/kg LPS (Sigma-Aldrich^®^, Germany), and after 24 h, all mice were euthanized to collect the intestine (the duodenum, the jejunum, the ileum, the cecum, and the rectum) samples. The body weights were documented daily. All of the experimental animals used in the current study were given standard feeding in the laboratory animal center of Hebei Agricultural University.

### Hematoxylin and eosin (H&E) staining

Intestinal samples from the mice of each group were collected primarily and preserved in paraformaldehyde (4.0%) for over 48 h followed by H&E staining from Pinuofei Biological Technology Co., Ltd (Wuhan, China). Olympus CX23 microscope (Olympus Co., Japan) was used for histological slide analysis. The villus height and crypt depth were recorded according to the previous study as depicted by Xu et al. (2021).

### DNA extraction and sequencing

Microbial DNA from the recta of each mouse group (*n* = 6) were retreived through fast DNA Stool Mini Kit (Qiagen, German) guided by the instructions. DNA products’ quantity and quality were detected *via* NanoDrop 2000 UV–vis spectrophotometer (Thermo Scientific, USA), and were examined through agarose gel electrophoresis (0.8%). The V3–V4 regions of bacteria 16S rRNA gene were amplified using primer pairs of 338F (5′-ACTCCTACGGGAGGCAGCAG-3′) and 806R (5′-GGACTA CHVGGG TWTCTA AT-3′) as reported in the previous study (Wang et al., 2019). Then, all the reaction products were purified and quantified using commercial AxyPrep DNA Gel Extraction Kit (Axygen Biosciences, USA) and QuantiFluor™-ST (Promega, USA), respectively, according to the instructions, followed by sequencing *via* the Illumina MiSeq platform (Bioyi Biotechnology Co., Ltd., China).

### Gut microbiota analysis

First, DADA2 and QIIME2^1^ were utilized to get cleaned results, amplicon sequence variant (ASV) (Callahan et al., 2016), and the taxonomy table (Bokulich et al., 2018). Alpha diversity analysis among different mouse groups were performed by calculating metrics such as Chao1, Observed species, Shannon, Faith’s PD, Pielou’s evenness, and Good’s coverage, as described in the previous study (Chen X. et al., 2022). Beta diversity analysis was carried out through the analysis of principal coordinate, monomeric multidimensional scaling (Vazquez-Baeza et al., 2013), unweighted pair-group method with arithmetic, and partial least squares discriminant. Different abundance among mice groups were explored by piloting methods of ANCOM, ANOVA, Kruskal Wallis, LEFSe, and DEseq2 (Segata et al., 2011; Love et al., 2014; Mandal et al., 2015). Finally, the potential functional profiles of KEGG Ortholog of mice gut microbiota were predicted through PICRUSt annotating MetaCyc and ENZYME database (Langille et al., 2013).

### Statistical analysis

All the currently obtained data were evaluated through ANOVA and Student’s *t*-test by employing IBM SPSS (20.0). Data were presented as means ± SD and were considered statistically significant when the *p*-value was < 0.05.

## Results

### Polysaccharide concentration, mice weights, and intestinal H&E examination

**FIGURE 1:**
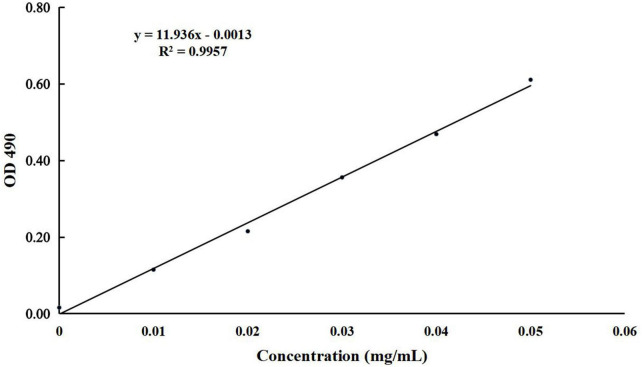
The standard curve of glucose used for detecting the concentration of BLBP.

**FIGURE 2:**
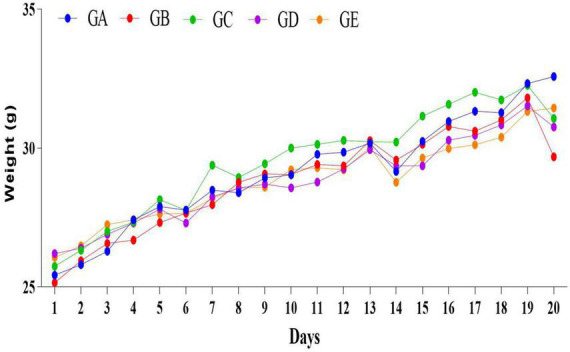
The daily weights of mice in different groups.

**FIGURE 3:**
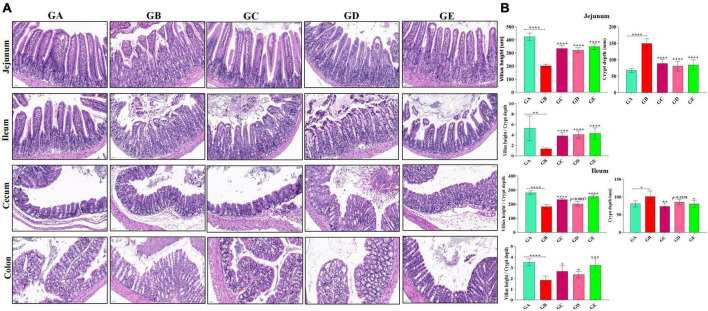
Effects of BLBP on the intestine of LPS-induced mice. **(A)** H&E staining analysis, **(B)** villus height, crypt depth, and villus height/crypt depth ratio. Scale bar 50 μm. Significance is presented as *p < 0.05, **p < 0.01, ***p < 0.001, and ****p < 0.0001; data are presented as the mean ± SEM (*n* = 6).

The concentration of present BLBP was 24.97% by the phenol-sulfuric method (Figure 1). There was no significant difference in the average daily weight of mice in different groups, the weight losses were slightly lower in groups GC and GE caused by the LPS challenge (Figure 2). An H&E examination revealed that the integrity of intestinal villi was destroyed by LPS, while the BLBP supplement alleviated the intestine damage. In the jejunum, the villus height in mice with the LPS challenge was significantly shorter (*p* < 0.0001), while the BLBP supplementation improved the villus height in mice in groups GC (*p* < 0.0001), GD (*p* < 0.0001), and GE (*p* < 0.0001). The crypt depth of the intestine of mice in group GB was significantly higher than that of mice in group GA (*p* < 0.0001) but decreased in the BLBP-supplemented groups (*p* < 0.0001). LPS significantly decreased the villus height and crypt depth of the intestine of mice in group GB (*p* < 0.01), while the BLBP supplementation significantly increased the villus height and the crypt depth of the intestine of mice in groups GC (*p* < 0.0001), GD (*p* < 0.0001), and GE (*p* < 0.0001). Similar results were found in the ileum of mice, and LPS challenge evidently decreased the villus height (*p* < 0.0001) and villus height/crypt depth (*p* < 0.0001) but increased crypt depth (*p* < 0.05) of the intestine of mice in group GB. Mice in groups GC and GE showed higher villus height (*p* < 0.0001) and villus height/crypt depth (*p* < 0.05) but lower crypt depth (*p* < 0.05; Figure 3).

### BLBP partly restored gut microbiota in mice induced by LPS

**FIGURE 4:**
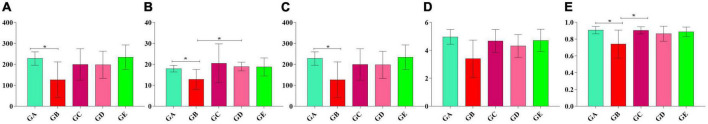
Alpha diversity analysis of mice gut microbiota in different groups. **(A)** chao1, **(B)** faith_pd, **(C)** observed_otus, **(D)** shannon, **(E)** simpson. Significance is presented as *p < 0.05; data are presented as the mean ± SEM (*n* = 6).

**FIGURE 5:**
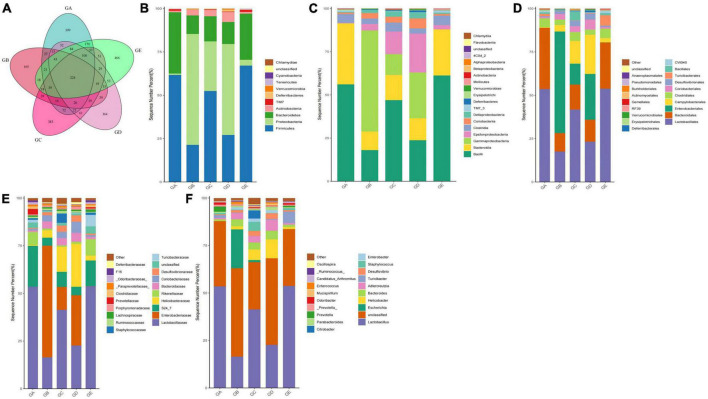
BLBP partly restored the gut microbiota in mice induced by LPS in different taxa. **(A)** Venn diagram, **(B)** Phylum, **(C)** Class, **(D)** Order, **(E)** Family, **(F)** Genus.

**FIGURE 6:**
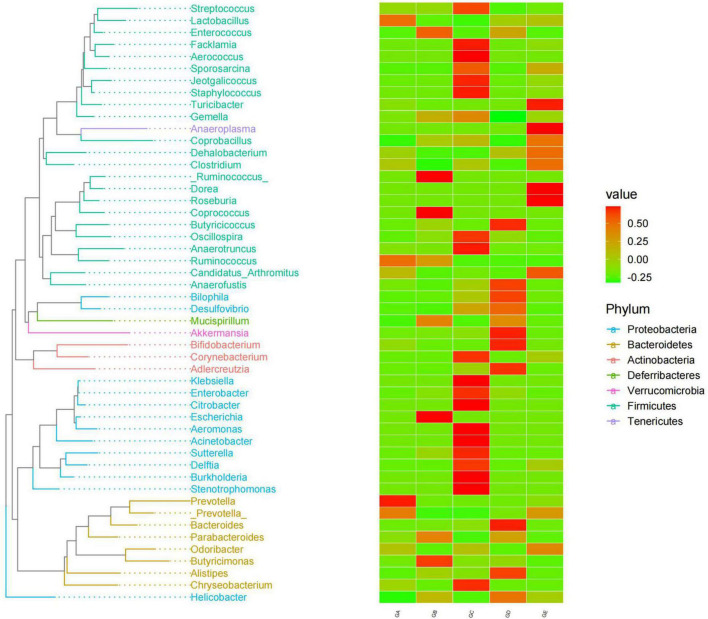
BLBP partly restored the gut microbiota in mice induced by LPS through phylogenetic analysis of the top 50 abundance-specific genera.

A total of over 59,000, 40,000, 50,000, 45,000, and 55,000 raw sequences were found in groups GA, GB, GC, GD, and GE, respectively (Table 1). More than 32,000 filtered sequences and 290,000 non-chimeric sequences were detected in all mice samples, respectively. Alpha diversity analysis was performed by examining the diversity indexes and revealed that chao1 (*p* < 0.05), faith_pd (*p* < 0.05), observed_otus (*p* < 0.05), and Simpson (*p* < 0.05) indices in group GB were lower as compared to group GA, while a non-significant difference was observed between different BLBP supplemented groups and the negative control group (GA; Figure 4). A total of 224 shared ASVs were found among all mouse groups (Figure 5A), and then, ASVs in all mouse groups were utilized for taxa analysis. At the phylum level, the dominant phyla in mice groups were *Firmicutes* (61.69%) and *Bacteroidetes* (35.30%) in GA, *Proteobacteria* (63.93%) and *Firmicutes* (21.40%) in GB, *Firmicutes* (52.46%) and *Proteobacteria* (28.58%) in GC, *Proteobacteria* (52.47%) and *Firmicutes* (27.03%) in GD, *Firmicutes* (67.04%) and *Bacteroidetes* (26.61%) in GE (Figure 5B). At the class level, *Bacilli* (56.06%) and *Bacteroidia* (35.30%) in GA, *Gammaproteobacteria* (58.58%) and *Bacilli* (18.05%) in GB, *Bacilli* (46.98%), *Bacteroidia* (14.44%), and *Epsilonproteobacteria* (13.00%) in GC, *Gammaproteobacteria* (26.47%), *Bacilli* (23.77%), and *Epsilonproteobacteria* (22.50%) in GD, and *Bacilli* (61.24%) and *Bacteroidia* (26.61%) in GE were mainly found (Figure 5C). At the order level, the primary orders in group GA were *Lactobacillales* (53.56%) and *Bacteroidales* (35.30%), in group GB were *Enterobacteriales* (58.58%), *Lactobacillales* (17.49%), and *Bacteroidales* (10.67%), in group GC were *Lactobacillales* (41.72%), *Bacteroidales* (14.44%), and *Enterobacteriales* (12.06%), in group GD were *Enterobacteriales* (26.45%), *Lactobacillales* (23.17%), and *Campylobacterales* (22.50%), and in group GE were *Lactobacillales* (53.80%) and *Bacteroidales* (26.61%; Figure 5D). At the family level, the highest abundance of *Lactobacillaceae* was found in groups GA (53.45%), GC (41.32%), and GE (53.72%), while the staple family in groups GB (58.58%) and GD (26.45%) was *Enterobacteriaceae* (Figure 5E). At the genus level, *Lactobacillus* and unclassified were the highest genera in groups GA, GC, GD, and GE, while a higher abundance of *Escherichia* (20.37%) was found in group GB (Figure 5F). Phylogenetic analysis of the top 50 abundant specific genera by ggtree in R showed that a high abundance of *Helicobacter*, *Parabacteroides*, *Mucispirillum*, and *Enterococcus* was uncovered in groups GB and GD, while a relatively higher abundance of *Odoribacter* and *Clostridium* was revealed in the mouse of groups GC, GD, and GE (Figure 6).

**TABLE 1:** Statistical analysis of achieved sequencing data in different mouse groups.

Sample	Input	Filtered	Percentage of input passed filter	Denoised	Merged	Percentage of input merged	Non-chimeric	Percentage of input non-chimeric
GA1	63,518	52,788	83.11	51,140	48,049	75.65	41,184	64.84
GA2	70,383	58,598	83.26	56,536	52,415	74.47	42,536	60.44
GA3	65,407	55,278	84.51	54,381	52,610	80.43	44,904	68.65
GA4	67,595	56,673	83.84	55,277	51,915	76.80	43,781	64.77
GA5	70,226	57,526	81.92	55,733	50,941	72.54	43,814	62.39
GA6	59,353	49,720	83.77	48,754	47,133	79.41	43,647	73.54
GB1	47,056	37,466	79.62	36,817	36,090	76.70	35,370	75.17
GB2	49,570	39,563	79.81	38,596	36,867	74.37	32,336	65.23
GB3	40,060	32,259	80.53	32,032.0	31,546	78.75	29,605	73.90
GB4	40,097	32,142	80.16	32,010	31,576	78.75	30,293	75.55
GB5	51,260	41,434	80.83	41,137	40,258	78.54	36,698	71.59
GB6	65,278	53,865	82.52	52,462	49,989	76.58	46,328	70.97
GC1	54,052	44,080	81.55	43,954	43,706	80.86	43,458	80.40
GC2	50,408	42,836	84.98	42,734	42,637	84.58	42,576	84.46
GC3	61,439	51,018	83.04	50,007	47,725	77.68	45,773	74.50
GC4	73,822	62,373	84.49	60,681	56,859	77.02	50,261	68.08
GC5	56,800	48,910	86.11	48,332	46,101	81.16	36,420	64.12
GC6	60,271	50,898	84.45	49,273	45,954	76.25	41,306	68.53
GD1	48,879	41,834	85.59	41,620	41,372	84.64	41,087	84.06
GD2	73,194	59,711	81.58	58,002	55,555	75.90	50,254	68.66
GD3	45,688	37,169	81.35	36,899	36,541	79.98	35,531	77.77
GD4	51,880	42,255	81.45	41,627	40,960	78.95	40,422	77.91
GD5	47,825	40,581	84.85	40,386	40,154	83.96	40,100	83.85
GD6	557,06	46,155	82.85	45,867	45,650	81.95	45,621	81.90
GE1	64,545	52,667	81.60	50,798	46,695	72.34	39,087	60.56
GE2	62,331	52,077	83.55	50,911	49,371	79.21	45,434	72.89
GE3	55,632	45,789	82.31	45,399	44,600	80.17	40,529	72.85
GE4	69,403	57,363	82.65	56,036	52,382	75.48	44,511	64.13
GE5	67,795	56,662	83.58	55,828	54,091	79.79	52,073	76.81

### BLBP regulated remarkable species in gut microbiota in mice induced by LPS

**FIGURE 7:**
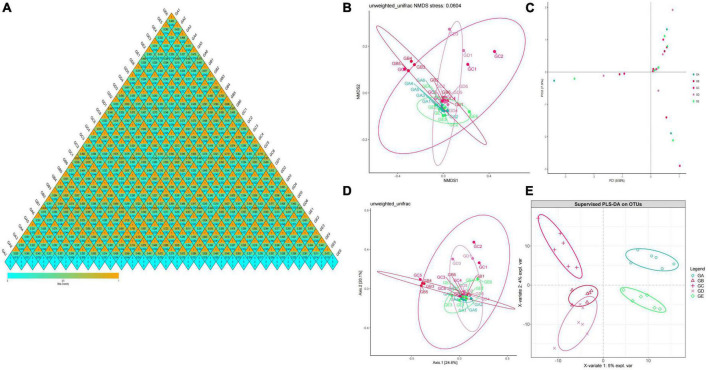
Beta diversity analysis of mice gut microbiota in different groups. **(A)** A heatmap of diversity index, **(B)** PCoA, **(C)** NMDS, **(D)** PLS-DA, **(E)** PCA.

**FIGURE 8:**
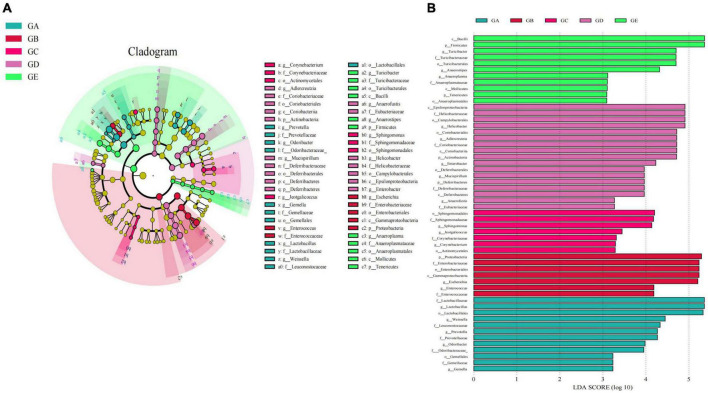
LEFSe analysis of significant difference species at genus level in mice gut microbiota. **(A)** Cladogram diagram, **(B)** LDA diagram.

Gut microbiota beta diversity analysis of mice in different groups through methods of heat map of diversity index (Figure 7A), PCoA (Figure 7B), NMDS (Figure 7C), PLS-DA (Figure 7D), and PCA demonstrated that species diversity difference in group GB compared with groups GA and GE, respectively (Figure 7E). To further reveal the remarkable species in mice gut microbiota, we performed a species comparison analysis. LEFSe analysis found that higher abundance of g_*Prevotella*, f_*Prevotellaceae*, g_*Odoribacter*, f_*Odoribacteraceae*, g_*Gemella*, f_*Gemellaceae*, o_*Gemellales*, g_*Lactobacillus*, f_*Lactobacillaceae*, g_*Weissella*, f_*Leuconostocaceae*, and o_*Lactobacillales* in mice in group GA, g_*Enterococcus*, f_*Enterococcaceae*, g_*Escherichia*, f_*Enterobacteriaceae*, o_*Enterobacteriales*, c_*Gammaproteobacteria*, and p_*Proteobacteria* in mice in group GB, g_*Corynebacterium*, f_*Corynebacteriaceae*, o_*Actinomycetales*, g_*Jeotgalicoccus*, g_*Sphingomonas*, f_*Sphingomonadaceae*, and o__*Sphingomonadales* in mice in group GC, g_*Adlercreutzia*, f_*Coriobacteriaceae*, o_*Coriobacteriales*, c_*Coriobacteriia*, p_*Actinobacteria*, g_*Mucispirillum*, f_*Deferribacteraceae*, o_*Deferribacterales*, c_*Deferribacteres*, p_*Deferribacteres*, g_*Helicobacter*, f_*Helicobacteraceae*, o_*Campylobacterales*, c_*Epsilonproteobacteria*, and g_*Enterobacter* in mice in group GD, and g_*Turicibacter*, f_*Turicibacteraceae*, o_*Turicibacterales*, c_*Bacilli*, g_*Anaerostipes*, p_*Firmicutes*, g_*Anaeroplasma*, f_*Anaeroplasmataceae*, o_*Anaeroplasmatales*, c_*Mollicutes*, and p_*Tenericutes* in mice in group GE (Figure 8A). Similar biomarkers were also detected by the LDA diagram (Figure 8B). The higher abundance of f_*Lactobacillaceae*, g_*Lactobacillus*, o_*Lactobacillales*, g_*Weissella*, f_*Leuconostocaceae*, g_*Prevotella*, f_*Prevotellaceae*, g_*Odoribacter*, f_*Odoribacteraceae*, o_*Gemellales*, f_*Gemellaceae*, and g_*Gemella* in mice in group GA, p_*Proteobacteria*, f_*Enterobacteriaceae*, o_*Enterobacteriales*, c_*Gammaproteobacteria*, g_*Escherichia*, g_*Enterococcus*, and f_*Enterococcaceae* in mice in group GB, o_*Sphingomonadales*, f_*Sphingomonadaceae*, g_*Sphingomonas*, g_*Jeotgalicoccus*, f_*Corynebacteriaceae*, g_*Corynebacterium*, and o__*Actinomycetales* in mice in group GC, c_*Epsilonproteobacteria*, f_*Helicobacteraceae*, o_*Campylobacterales*, g_*Helicobacter*, o_*Coriobacteriales*, g_*Adlercreutzia*, f_*Coriobacteriaceae*, c_*Coriobacteriia*, p_*Actinobacteria*, g_*Enterobacter*, o_*Deferribacterales*, g_*Mucispirillum*, p_*Deferribacteres*, f_*Deferribacteraceae*, c_*Deferribacteres*, g_*Anaerofustis*, and f_*Eubacteriaceae* in mice in group GD, and c_*Bacilli*, p_*Firmicutes*, g_*Turicibacter*, f_*Turicibacteraceae*, o_*Turicibacterales*, g_*Anaerostipes*, g_*Anaeroplasma*, f_*Anaeroplasmataceae*, c_*Mollicutes*, p_*Tenericutes*, and o_*Anaeroplasmatales* in mice in group GE.

**FIGURE 9:**
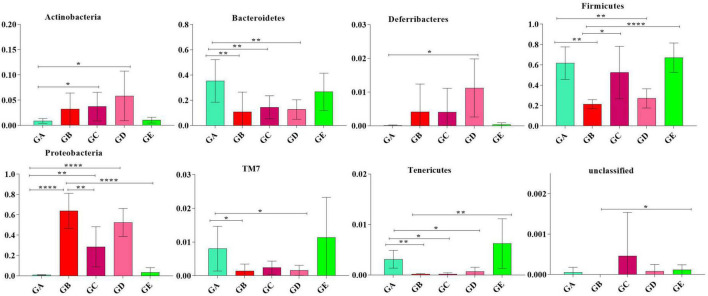
ANOVA analysis of remarkable species in gut microbiota at the phylum level in mice. Significance is presented as *p < 0.05, **p < 0.01, and ****p < 0.0001; data are presented as the mean ± SEM (*n* = 6).

**FIGURE 10:**
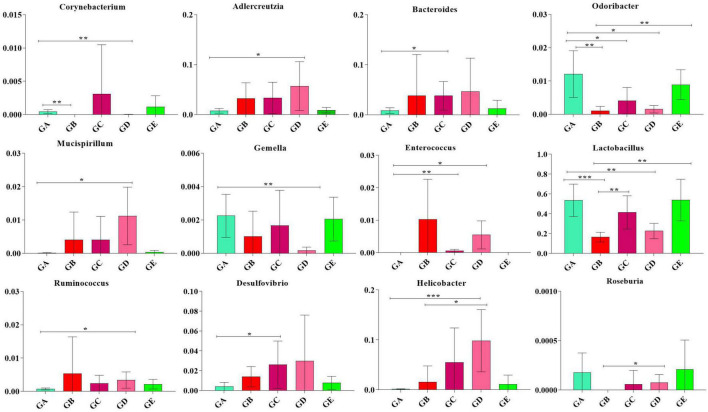
ANOVA analysis of remarkable species in gut microbiota at the genus level in mice. Significance is presented as *p < 0.05, **p < 0.01, and ***p < 0.001; data are presented as the mean ± SEM (*n* = 6).

To describe the effect of BLBP on regulating remarkable species in gut microbiota in mice, ANOVA analysis was performed. At the phylum level, the abundance of *Actinobacteria* in mice in group GA was significantly lower than that in mice in groups GC (*p* < 0.05) and GD (*p* < 0.05). The abundance of *Bacteroidetes* in mice in group GA was significantly higher than that in mice in groups GB (*p* < 0.01), GC (*p* < 0.01), and GD (*p* < 0.01). The abundance of *Deferribacteres* in mice in group GD was noticeably higher than in mice in group GA (*p* < 0.05). The abundance of *Firmicutes* in mice in groups GB (*p* < 0.01) and GD (*p* < 0.01) were significantly lower than that in mice in group GA, while the abundance of *Firmicutes* in mice in groups GC (*p* < 0.05) and GE (*p* < 0.0001) were significantly higher than that in mice in group GB. The abundance of *Proteobacteria* in mice in group GB was significantly higher than that in mice in groups GA (*p* < 0.0001), GC (*p* < 0.01), and GE (*p* < 0.0001). The abundance of TM7 in mice in group GA was significantly higher than that in mice in groups GB (*p* < 0.05) and GD (*p* < 0.05). The abundance of *Tenericutes* was found to be significantly lower in mice in groups GB (*p* < 0.01), GC (*p* < 0.05), and GD (*p* < 0.05) but higher in mice in group GE (Figure 9). At the genus level, *Corynebacterium* (*p* < 0.01), *Odoribacter* (*p* < 0.01), and *Lactobacillus* (*p* < 0.001) were significantly higher in mice in groups GA and GB, while *Butyricimonas* (*p* < 0.01) was higher only in mice in group GB (Figure 10).

### BLBP affected gut microbiota function in mice induced by LPS

**FIGURE 11:**
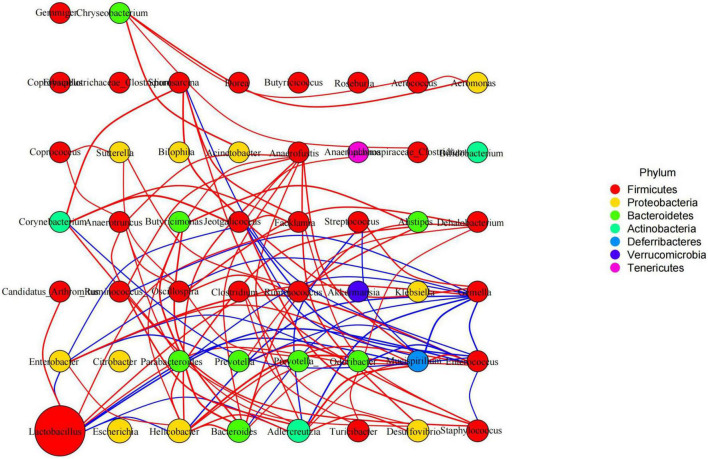
Species interaction network diagram of mice gut microbiota at the genus level. Red represents a positive correlation, blue represents a negative correlation.

**FIGURE 12:**
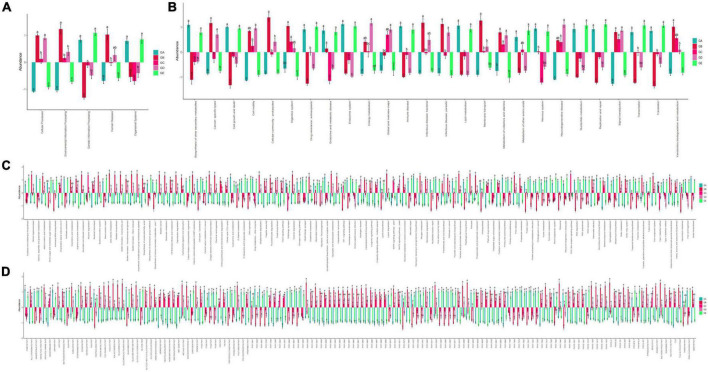
Function predicting analysis of mice gut microbiota. **(A)** KEGG L1, **(B)** KEGG L2, **(C)** KEGG L3, **(D)** MetaCyc.

Species interaction network diagram analysis found that the bacterial interaction was positively related to *Lactobacillus*, *Facklamia*, *Anaerofustis*, *Dehalobacterium*, and *Anaerotruncus* but negatively related to *Mucispirillum*, *Gemella*, *Jeotgalicoccus*, *Streptococcus*, and *Enterobacter* (Figure 11). KEGG L1 analysis revealed that the LPS challenge significantly affected the microbiota function of the cellular processes (*p* < 0.0001), the environmental information processes (*p* < 0.0001), the genetic information processes (*p* < 0.0001), the human diseases (*p* < 0.01), and the organismal systems (*p* < 0.01). The BLBP supplementation in mice in groups of GC and GE reversed the effect, especially in GE mice (Figure 12A). KEGG L2 analysis showed that the LPS significantly changed cell growth and death (*p* < 0.0001), cell motility (*p* < 0.0001), cellular community-prokaryotes (*p* < 0.0001), membrane transport (*p* < 0.001), signal transduction (*p* < 0.0001), folding, sorting, and degradation (*p* < 0.01), replication and repair (*p* < 0.0001), transcription (*p* < 0.0001), translation (*p* < 0.0001), drug resistance: antimicrobial (*p* < 0.01), drug resistance: antineoplastic (*p* < 0.0001), infectious disease: bacterial (*p* < 0.0001), amino acid metabolism (*p* < 0.05), biosynthesis of other secondary metabolites (*p* < 0.0001), carbohydrate metabolism (*p* < 0.01), energy metabolism (*p* < 0.001), glycan biosynthesis and metabolism (*p* < 0.05), lipid metabolism (*p* < 0.0001), and xenobiotic biodegradation and metabolism (*p* < 0.001), and the BLBP supplementation in groups GC and GE partly reversed the effect, especially in the GE group (Figure 12B). KEGG L3 analysis revealed that the significant changes of bacterial chemotaxis (*p* < 0.01), biofilm formation-*Escherichia coli* (*p* < 0.0001), bacterial secretion system (*p* < 0.0001), protein processing in the endoplasmic reticulum (*p* < 0.0001), DNA replication (*p* < 0.0001), bacterial invasion of epithelial cells (*p* < 0.01), and *Staphylococcus aureus* infection (*p* < 0.01) in mice induced by the LPS, while the BLBP supplementation partly reversed the effect, especially in GE mice (Figure 12C). MetaCyc analysis described that mice treated with the LPS showed visible differences in the signaling pathways of 1CMET2-PWY (*p* < 0.0001), ALL-CHORISMATE-PWY (*p* < 0.0001), ARG + POLYAMINE-SYN (*p* < 0.0001), and so forth, while the BLBP supplementation in mice in the GC and GE groups partly reversed the effect, especially in mice in the GE group (Figure 12D).

## Discussion

Many studies found that “microbiota repair” could alleviate diseases through complete fecal communities, probiotics, synbiotics, and herbal extracts (Blanton et al., 2016; Ahern and Maloy, 2020; Gomaa, 2020; Lv et al., 2022). *Lycium barbarum* is a popular fruit and functional medicinal plant (Zhao et al., 2020), which has anti-oxidative, anti-inflammatory, and anti-aging functions. Besides, it regulates gut microbiota (Mocan et al., 2014) and antagonizes the LPS-induced inflammation by possibly altering the glycolysis of macrophages (Ding et al., 2021). Intestinal inflammation and oxidative damage are widely detected in different diseases, whereas the functional characteristic of *Lycium barbarum* makes it a potential therapy for intestinal damage.

In the current study, we explored the effect of BLBP on the LPS-induced damage and intestinal flora disturbance in mice by piloting 16s rRNA sequencing, which is an important and widely utilized method in characterizing microbiota (Wong et al., 2022). The results showed that the BLBP supplement could regulate the weight losses caused by the LPS, especially in mice in groups GC and GE (Figure 2). Intestinal integrity damage and broken villus with significantly shorter villus heights and higher crypt depths were seen in LPS-exposed mice similar to what was reported by previous studies (Duan et al., 2018; Wang et al., 2021; Chen X. et al., 2022). It was found that the treatment with BLBP could mediate intestinal damage in mice, especially in mice from groups GC and GD (Figure 3). To uncover the potential relationship with the intestinal microbiota, we performed 16s rRNA sequencing and achieved a total of 1,689,473 raw and 1,399,690 filtered sequences in this study. LPS-induced changes also decreased the alpha diversity indexes (*p* < 0.05), while no significant differences were found among the different BLBP treatment groups (Figure 4), which demonstrated that the BLBP could reverse the decreasing trend of species diversity caused by LPS. Further analysis revealed that the BLBP supplement could partly restore the primary and remarkable species in gut microbiota in mice induced by LPS through analysis of ASVs taxa (Figure 5), phylogenetic tree (Figure 6), and LEFSe (Figure 8). Those results revealed that the BLBP could relieve intestinal damage through “microbiota repair.” Alteration of intestinal flora will affect its function; hence, we preformed the functional predicting analysis of mice and found that the LPS changed 282 metabolic pathways in KEGG L2, 77 metabolic pathways in KEGG L3, and 205 metabolic pathways in MetaCyc, respectively; the reverse effects on those metabolic pathways were watched in mice with the treatment of BLBP, especially in mice in group GE (Figure 12).

In total, 8 phyla and 13 genera were found among the current five mouse groups through ANOVA analysis (Figures 9, 10). Cani and Delzenne (2010) reported that gut microbiota is critical for numerous aspects of host physiology and contributes effectively to regulating metabolism, development, and immunity besides their potent role in regulating inflammation and pain. In comparison to bacterial species (genus) content, *Adlercreutzia* was identified with the LPS-induced mice in this study. The high concentration of BLBP-treated mice in group GD showed a similar abundance of *Adlercreutzia*. Jin and Zhang (2020) reported lower proportions of *Adlercreutzia* in high-fat diet mice, proposing its role in intestinal susceptibility toward carcinogens. However, Dekker Nitert et al. (2020) reported the presence of *Adlercreutzi* in the intestine along with back pain issues, showing a robust relationship with the pathogenesis of back pain. It is assumed that the mere presence or absence of *Adlercreutzia* rather than its abundance (% age) drives the impact associated with the presence of this particular genus. A slight increase in *Bacteroides*, *Mucispirillum*, opportunistic pathogenic *Enterococcus*, *Ruminococcus*, and pro-inflammatory bacteria of *Desulfovibrio* and *Helicobacter* wwas found in LPS-induced mice, which was in accordance with the results found in dogs with mammary tumor (Zheng et al., 2022), mice with colitis (Lee et al., 2022), patients with atrial fibrillation (Huang et al., 2022), people with obseity (Xu et al., 2022), and in high-fat diet-induced obesity mice. Mice in group GE demonstrated a decrease in those genera to the same level as control in mice in group GA, which may explain that BLBP mediates intestinal damage *via* a decrease of those relatively negative bacteria in mice. The abundance of *Gemella* and beneficial bacteria of *Roseburia* in presently used mice were slightly lower in LPS-induced mice, which was in line with infants with food allergy and atopic dermatitis (Łoś-Rycharska et al., 2021) and ulcerative colitis rats (Ran et al., 2022), respectively, and similar to the effect of Chinese dwarf cherry fermentation juice on ulcerative colitis rats, mice treated by BLBP show higher abundance of *Gemella* and *Roseburia*. As mentioned earlier, a lower abundance of *Corynebacterium* was examined in *Eimeria*-infected birds and a supplement of probiotic *bacillus subtilis* could increase the abundance of *Corynebacterium* (Memon et al., 2022), which was in line with the current study and found to increase *Corynebacterium* in BLBP-treated mice with the LPS challenge. Previous study found increased *Odoribacter* in fecal microbiota transplantation-treated colitis mice (Zhang et al., 2020), which was in line with LPS-induced mice, and higher concentration treatment of BLBP depicted a higher abundance of *Odoribacter* in mice in group GE (*p* < 0.01). *Lactobacillus* is a commonly recognized genus of probiotics, which can maintain the intestinal barrier and provide protection against inflammation (Bai et al., 2022), and a lower abundance of *Lactobacillus* had been found in antibiotic-associated diarrhea in mice (Chen C. et al., 2022) and ulcerative colitis mice (Hu et al., 2022). In the current study, a higher abundance of *Lactobacillus* was detected in mice in all BLBP-supplemented groups, especially in group GC and GE, which was in accordance with *Lycium barbarum* arabinogalactan treated mice with colitis (Cao et al., 2022). The current study may indicate that the LPS challenge caused intestinal damage by decreasing the abundance of *Corynebacterium*, *Odoribacter*, and *Lactobacillus* while BLBP could alleviate it by increasing the abundance of bacteria from those genera.

## Conclusion

In conclusion, the current study shows that BLBP can effectively attenuate intestine damage through the regulation of intestinal microbiota, and *Corynebacterium*, *Odoribacter*, and *Lactobacillus* are important links. These findings may provide new insights in the prevention of intestinal disease using food and medicine homologous of *Lycium ruthenicum*. This is an exploratory study of gut microbiota influenced by BLBP intake; however, further investigations are needed to confirm our findings and to clarify the potential mechanism by which BLBP attenuates intestinal damage other than microbiota regulation to establish its role as a therapeutic agent.

## Data availability statement

The datasets presented in this study can be found in online repositories. The names of the repository/repositories and accession number(s) can be found below: https://www.ncbi.nlm.nih.gov/, PRJNA889725.

## Ethics statement

All the experiment operations were under the instructions and approval of Laboratory Animals Research Centre and the Ethics Committee of Hebei Agriculture University.

## Author contributions

AY and YS: research idea and methodology. AY, HD, and JL: reagents, materials, and analysis tools. AY, CB, ZH, and ZW: writing – original draft and preparation. YS, SN, and SL: writing – review and editing. YS and SL: visualization and supervision. All authors are known and approved the final article.

## Footnotes

1
https://github.com/QIIME2/q2-feature-classifier
